# Wild waterfowl migration and domestic duck density shape the epidemiology of highly pathogenic H5N8 influenza in the Republic of Korea

**DOI:** 10.1016/j.meegid.2015.06.014

**Published:** 2015-08

**Authors:** Sarah C. Hill, Youn-Jeong Lee, Byung-Min Song, Hyun-Mi Kang, Eun-Kyoung Lee, Amanda Hanna, Marius Gilbert, Ian H. Brown, Oliver G. Pybus

**Affiliations:** a Department of Zoology, University of Oxford, Oxford OX1 3PS, United Kingdom; b Animal and Plant Quarantine Agency (QIA), 175 Anyangro, Anyangsi, Gyeonggido 430-757, Republic of Korea; c Department of Virology, Animal and Plant Health Agency (APHA), Weybridge KT15 3NB, United Kingdom; d Biological Control and Spatial Ecology, Université Libre de Bruxelles, B-1050 Brussels, Belgium; e Fonds National de la Recherche Scientifique, 1080 Molenbeek-Saint-Jean, Belgium

**Keywords:** ROK, Republic of Korea, Avian influenza, H5N8, Phylogeography, Phylogenetics, Ecology, Korea

## Abstract

Highly pathogenic avian influenza (HPAI) viruses threaten human and animal health yet their emergence is poorly understood, partly because sampling of the HPAI Asian-origin H5N1 lineage immediately after its identification in 1996 was comparatively sparse. The discovery of a novel H5N8 virus in 2013 provides a new opportunity to investigate HPAI emergence in greater detail. Here we investigate the origin and transmission of H5N8 in the Republic of Korea, the second country to report the new strain. We reconstruct viral spread using phylogeographic methods and interpret the results in the context of ecological data on poultry density, overwintering wild bird numbers, and bird migration patterns. Our results indicate that wild waterfowl migration and domestic duck density were important to H5N8 epidemiology. Specifically, we infer that H5N8 entered the Republic of Korea via Jeonbuk province, then spread rapidly among western provinces where densities of overwintering waterfowl and domestic ducks are higher, yet rarely persisted in eastern regions. The common ancestor of H5N8 in the Republic of Korea was estimated to have arrived during the peak of inward migration of overwintering birds. Recent virus isolations likely represent re-introductions via bird migration from an as-yet unsampled reservoir. Based on the limited data from outside the Republic of Korea, our data suggest that H5N8 may have entered Europe at least twice, and Asia at least three times from this reservoir, most likely carried by wild migrating birds.

## Highlights

• Phylogeographic analyses of H5N8, including 49 new sequences from South Korea.
• H5N8 movement was mostly among areas dense in wild and domestic ducks.
• New viral introductions to South Korea occurred at time of wild bird migration.
• H5N8 epidemiology is shaped by wild waterfowl migration and domestic duck density.
• H5N8 may have entered Europe at least twice, and Asia at least three times.

## 1 Introduction

Highly pathogenic avian influenza (HPAI) viruses are a threat to human and animal health and cause considerable economic damage. H5N1 viruses of the A/goose/Guangdong/1/96 (GsGd) lineage have become endemic in parts of Asia (including Bangladesh, China, India, Indonesia and Vietnam) and in Egypt, and have resulted in the culling of over 250 million birds worldwide (Swayne, 2012; Xu et al., 1999). Previous research has tried to characterize where GsGd lineage H5N1 emerged and how it subsequently spread (Chen et al., 2006; Claas et al., 1998; Guan et al., 1999; Lemey et al., 2009; Li et al., 2004; Matrosovich et al., 1999; Shortridge et al., 1998; Suarez et al., 1998; Xu et al., 1999). Unfortunately, comparatively poor sampling in the months immediately following the identification of GsGd lineage H5N1 in 1996 has prevented an accurate reconstruction of its emergence.

Since 2009, there has been an unprecedented surge in the emergence of novel reassortant H5 viruses of the GsGd lineage (de Vries et al., 2015; Liu et al., 2013; Wong et al., 2015; Zhao et al., 2012), most notably including H5N8 (Zhao et al., 2013). The emergence of these novel H5 viruses should be easier to investigate than that of GsGd lineage H5N1 because more viral genetic data is available from the early phase of emergence. These novel viruses thus provide an fresh opportunity to investigate in detail the factors behind avian influenza virus emergence (Verhagen et al., 2015a).

The first case of GsGd lineage H5N8 (henceforth referred to as H5N8) in Asia was reported in China in 2010 (Zhao et al., 2013). In November 2013, several viruses (clade 2.3.4.4; Donis et al., 2015) were isolated from domestic and wild ducks in China, which appeared to be novel reassortants of the 2010 H5N8 virus (Fan et al., 2014; Lee et al., 2014; Wu et al., 2014). By January 2014, the first outbreaks outside of China were noted in the Republic of Korea (ROK; commonly known as South Korea) (Lee et al., 2014). H5N8 viruses were found in domestic ducks and wild Anseriformes around the Donglim Reservoir (Jeonbuk province), an important habitat for wild migratory birds (Jeong et al., 2014; Yoon et al., 2015). During 2014, outbreaks of H5N8 were confirmed on at least 33 different farms in ROK. A total of 296 H5N8 viruses were isolated in ROK, including 43 from wild birds and 253 from poultry farms. By the beginning of 2015 the H5N8 virus had been reported in wild and domestic bird populations throughout Asia, Russia, Europe, and, most notably, in North America.

The HPAI H5N1 and H5N2 viruses discovered in North America in December 2014 and January 2015 appear to be reassortants of the Eurasian H5N8 virus, carrying an HA segment of the GsGd lineage (Pasick et al., 2015; USDA APHIS, 2015). Eurasian H5 viruses have never been identified before in North American birds (Ip et al., 2015; Jhung and Nelson, 2015), so the appearance of Eurasian-like viruses in North America represents an unexpected shift in the global epidemiology of avian influenza.

Wild birds, particularly Anseriformes and Charadriiformes, are considered the primary natural reservoir for low pathogenicity avian influenza viruses (Olsen et al., 2006; Olson et al., 2014). In contrast, the relative contribution of wild birds versus domestic poultry to HPAI virus persistence, and to its introduction to new locations, remains unresolved (Olsen et al., 2006). It has been suggested that H5N8 may be maintained and spread by wild birds more readily than GsGd lineage H5N1 viruses (European Food Safety Authority, 2014; Kang et al., 2015), a difference that might in part explain the recent, notable changes in H5 epidemiology. For at least some species of wild birds, infection symptoms may be mild enough to allow migration whilst infected with H5N8 (Jeong et al., 2014; Kang et al., 2015; Kim et al., 2015). Recent studies have found that H5N8 viruses isolated in Europe, Russia and Asia are surprisingly genetically similar given the large intercontinental distances separating the locations from which they were sampled (Bouwstra et al., 2015; Dalby and Iqbal, 2015; Hanna et al., 2015; Harder et al., 2015; Ozawa et al., 2015; Verhagen et al., 2015b). These observations have prompted suggestions that migrating birds may have recently carried H5N8 from a common location (Bouwstra et al., 2015; Dalby and Iqbal, 2015; Verhagen et al., 2015b). Testing this hypothesis is of practical importance, as current surveillance measures were largely developed in the context of GsGd lineage H5N1 and may need adapting for H5N8 if transmission pathways are different.

Phylogeographic analyses can be used to reconstruct the geographic dispersal of a virus lineage from viral genome sequences (Holmes, 2004). Such analyses have been usefully applied to many rapidly-evolving viruses, including GsGd lineage H5N1 (e.g., (Lemey et al., 2009)). Ecological data, for example on poultry, human or waterfowl population densities, can be used to identify risk factors for the emergence of avian influenza (e.g., H7N9 in China (Gilbert et al., 2014), H5N1 in China (Martin et al., 2011)), and can be combined with genetic sequence data from outbreaks (Magee et al., 2014; Ypma et al., 2012). Although interpretation of phylogeographic analyses in the context of known ecology could help illuminate the causes of HPAI virus emergence and spread, no such studies have been attempted for H5N8, probably because insufficient sequences from any one affected region have been available for analysis.

Poultry production in ROK (including both duck and chicken farming for meat and eggs) has rapidly increased over the past 15 years. Census counts suggest over 150 million chickens and over 8 million ducks are farmed commercially in ROK (Republic of Korea Livestock Census 2014; http://kostat.go.kr) under high to moderate levels of biosecurity. Poultry are also produced for trading in live bird markets on small scale farms with low to moderate levels of biosecurity. Many species of wild birds regularly migrate to ROK to overwinter, creating distinct temporal dynamics in the available hosts for influenza viruses. Integrating virus genetic information with ecological information on host availability is critical for understanding the spread of avian influenza virus in the country.

Here we investigate the geographic spread of H5N8 with a particular focus on the Republic of Korea. ROK was one of the first countries to report outbreaks in both the 2003 GsGd lineage H5N1 outbreak and the ongoing H5N8 outbreak. We report 49 new sequences of the HA segment of H5N8 isolates from the country. Phylogeographic methods are used to reconstruct the spatial spread of the virus and to investigate how this dispersal was shaped by bird density and migration patterns. Understanding how H5N8 became established in ROK could help inform strategies to prevent future epidemics and to mitigate the risks arising from a greater diversity of HPAI viruses in poultry worldwide.

## 2 Materials and methods

### 2.1 Bird ecology data

#### 2.1.1 Longitudinal data on bird counts and waterfowl numbers

**Fig. 1:**
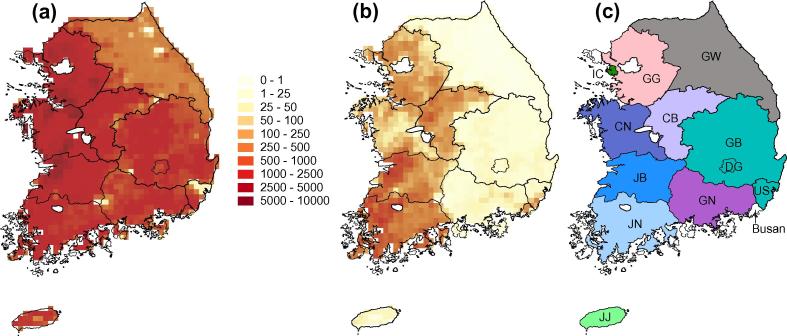
Maps showing domestic poultry density (number per kilometer, colors in key) in ROK according to the Gridded Livestock of the World 2.0 (Robinson et al., 2014). (a) Domestic chicken density. (b) Domestic duck density. (c) Map of provinces. Colors correspond to the branch color scheme used in Fig. 3. Province abbreviations are as follows; CB: Chungbuk, CN: Chungnam, DG: Daegu, GB: Gyeongbuk, GG: Gyeonggi, GN: Gyeongnam, GW: Gangwon, IC: Incheon, JB: Jeonbuk, JJ: Jeju, JN: Jeonnam, US: Ulsan. (For interpretation of the references to color in this figure legend, the reader is referred to the web version of this article.)

Ecological information on seasonal and overwintering wild waterfowl counts was collated. Data was only obtained on wild birds from the family *Anatidae*, as the H5N8 virus has been mostly isolated from that family. A literature review was undertaken to obtain longitudinal bird count data, under the conditions that the data must include waterfowl counts taken for at least one year, with sampling at least every month (Web of Science search terms ‘Korea AND (longitudinal OR temporal OR seasonal OR changes OR dynamics) AND (bird OR waterfowl OR duck)’, with papers in English and published after January 2000 considered). Longitudinally sampled waterfowl counts were obtained for four different sites in ROK: (i) Mokpo Namhang Urban Wetland, Jeonnam province (daily waterfowl counts April 2006-July 2010; Birds Korea), (ii) Sihwa Lake, Gyeongii province (monthly waterfowl counts January to December 2009; Park et al., 2011), (iii) Nakdonggang Estuary, Busan province (monthly *Mergus albellus*, *Mergus serrator* and *Bucephala clangula* count, 2002–2008; (Hong, 2013; Hong and Lee, 2012), and (iv) Junam Reservoir, near Changwon, Gyeongnam province. Province locations are shown in Fig. 1. Waterfowl counts were scaled so maximum count at each site is equal to one. Data were plotted using R version 3.1.1 (R Core Team, 2014) and the package ggplot2 (Wickham, 2009), as implemented in RStudio (RStudio, 2013).

Maps of estimated numbers of the four most common waterfowl in ROK were obtained from the ROK Ministry of Environment Wild Bird Census for Winter 2014 report Kim et al. (2014a). The Ministry simultaneously observed 195 national wildlife reserves for migratory birds from January 24th to 26th. At each reserve, bird numbers were counted by two specialists using line and point census methods. For the line census, researchers counted birds whilst walking along roads. For the point census, total bird numbers were counted using binoculars from a single observation point, usually on the water.

#### 2.1.2 Poultry density and temporal data

Maps of livestock distributions for the Republic of Korea were produced for domestic ducks and chickens, using the Gridded Livestock of the World version 2 (GLW version 2; Robinson et al., 2014). Briefly, the GLW version 2 uses sub-national statistics and predictor variables of livestock density to model the density of livestock at a 1 km by 1 km scale. Data on seasonality of poultry counts throughout the year (not shown) were obtained from the Republic of Korea Livestock Census 2014 (published by Statistics Korea; http://kostat.go.kr). The census is based on poultry counts for a single day in each quarter, for all farms breeding >3000 chickens or >2000 ducks.

### 2.2 Sequence data

In total, 296 H5N8 viruses were isolated in ROK in 2014. The HA genes of 49 H5N8 isolates from ROK were sequenced to complement previously published sequences. Isolates were chosen for sequencing in order to generate a dataset that, when combined with previously published sequences, included at least 4 strains per month and 1 strain per province (Table A.1). Viral RNA was extracted from the allantoic fluid of embryonated eggs using the Viral Gene-spin viral DNA/RNA extraction kit (iNtRON) according to the manufacturer’s instructions. The HA gene was amplified with gene-specific universal primers (Hoffmann et al., 2001), using the One Step RT-PCR Kit (Qiagen). PCR products were purified from agarose gels using the Qiaquick gel extraction kit (Qiagen). Full genomic DNA was sequenced by COSMO genetech (Seoul, South Korea) with an ABI 3730 genetic analyzer (Applied Biosystem). Contig assembly was performed using CLC Main Workbench Ver. 6.8.2 (CLC bio). GISAID accession numbers for these new sequences are EPI573192, EPI573195–EPI573242 (Table A.2).

In addition to the 49 new sequences, 51 HA clade 2.3.4.4 sequences were downloaded from the Influenza Research Database (FluDB), and 22 HA sequences were downloaded from the Global Initiative on Sharing Avian Influenza Data (GISAID) EpiFlu. This resulted in a dataset of 122 HA sequences, of which 88 were from ROK (see Table A.2 for details). Acknowledgements of originating and submitting laboratories for GISAID sequences are provided in Table A.2. Geographic locations, collection dates and hosts are also provided. All sequences were combined and aligned using Muscle 3.8.31 (Edgar, 2004) as implemented in Mega 6.0 (Tamura et al., 2013). The alignment was trimmed to coding regions only.

### 2.3 Bayesian molecular clock phylogeography

#### 2.3.1 Model selection

Initial maximum likelihood trees were generated for the HA segment using Garli 2.01 (Zwickl, 2006). Path-O-Gen (Rambaut, 2009) was used to confirm strong temporal signal and the appropriateness of a molecular clock approach. Coalescent and nucleotide substitution models were subsequently chosen following model comparison in BEAST (Drummond et al., 2012; Drummond and Rambaut, 2007). All combinations of the following models were compared: constant size versus Bayesian skyline (five groups) coalescent models and SRD06 versus General Time Reversible nucleotide substitution models, with gamma distributed rate heterogeneity. The SRD06 nucleotide substitution model and Bayesian skyline coalescent tree prior were chosen for all subsequent analyses based on model comparison using the harmonic mean estimator and Akaike information criteration, as implemented in BEAST.

Early results obtained during model selection suggested that maximum clade credibility (MCC) trees included a clade of European, Russian and Asian samples (clade C4 in Fig. 3b), within which internal nodes were poorly supported. In an attempt to clarify this portion of the tree topology, available NA sequences for clade C4 strains were also included (see below; Table A.2). Maximum likelihood trees were generated using Garli 2.01 (Zwickl, 2006), and a concatenated alignment of HA and NA sequences was scanned for recombination using GARD (Delport et al., 2010; Pond et al., 2005, 2006) to check that no isolates within clade C4 had undergone reassortment in the NA segment.

A prior distribution for the rate of molecular evolution was chosen based on a preliminary analysis in BEAST. Specifically, 30 sequences from Europe and Asia spanning the period 2007–2014 were downloaded from the Influenza Research Database for both the HA (H5, clades 2.2 and 2.3) and NA segments (N8). For both segments, the 30 reference sequences and available H5N8 sequences were combined and aligned using Muscle, as implemented in Mega 6.0 (Edgar, 2004; Tamura et al., 2013). BEAST was used to generate estimates of the nucleotide substitution rate for the HA and NA segments from these temporally-structured alignments using the relaxed lognormal molecular clock, which were then used as informative priors of the substitution rate parameters in subsequent analyses.

#### 2.3.2 Phylogeographic analyses

Discrete phylogeographic analyses were performed to reconstruct the geographic spread of the virus, whilst simultaneously estimating the phylogenetic tree. These analyses were implemented in BEAST (Drummond et al., 2012; Drummond and Rambaut, 2007; Lemey et al., 2009), using the BEAGLE library (Ayres et al., 2011). An asymmetric model was chosen to allow different rates of lineage movement in opposite directions between each pair of locations (Edwards et al., 2011). Analyses were performed under the SRD06 substitution model, using an uncorrelated lognormal relaxed clock and Bayesian skyline coalescent model. For most isolates, only the HA segment sequences were used, but for sequences in clade C4 NA sequences were also included, where available. Molecular clock and substitution models were unlinked for the two segments, whilst the phylogeny itself was linked. Sequences were coded by area (Fig. 1). A fully annotated MCC phylogeny is provided in Fig. A.1.

Discrete phylogeographic analyses were conducted both with and without Bayesian stochastic search variable selection (BSSVS). BSSVS identifies which geographic links are strongly supported by the data, by assuming that for many possible pairs of locations there will be limited or no observations of viral lineage movement (Lemey et al., 2009). Between two and four independent runs of 200 million steps of a Markov chain were performed for each analysis, with sampling every 20,000 steps. Convergence of the runs and effective sample sizes were checked using Tracer (http://tree.bio.ed.ac.uk). Runs were combined after discarding 20 million steps as burnin. MCC trees were generated using TreeAnnotator and visualized using Figtree (http://tree.bio.ed.ac.uk).

A reduced dataset was generated to exclude sequences that were descended from specifically identified long branches in the MCC tree (see Section 3.3), reflecting our view that these branches represent virus re-introduction from outside ROK, not cryptic persistence within ROK throughout 2014. Using a subsampled empirical tree distribution of 1000 trees generated during discrete trait reconstruction with BSSVS on this reduced dataset, Markov jump analysis (Minin and Suchard, 2008; O’Brien et al., 2009; Talbi et al., 2010) was performed to estimate the number of jumps between pairs of locations, for each with a Bayes Factor >10. SPREAD was then used to calculate Bayes Factor support for the location rate indicators (Bielejec et al., 2011). These values give an indication of the intensity of movement among locations.

Heterogeneous sampling may affect phylogenetic inference, with less well-sampled locations tending to be inferred as sink regions. Because H5N8 sequence data is relatively limited, it was not possible to use downsampling approaches (e.g., Faria et al., 2014) to create a dataset with equal sample sizes for each region. To assess the effects of including locations for which only one sequence is available, we re-ran the phylogeographic analysis with BSSVS whilst (i) removing locations with only one sequence from the dataset, or (ii) combining locations with only one sequence with the geographically closest province (as per the case map in Yoon et al., 2015; Fig. A.2).

To further investigate the phylogenetic structure of clade C4, monophyly statistics and TMRCAs for defined groups of viruses within this clade were estimated. Both HA and NA sequences were included where available for this clade (Table A.2). Statistics were estimated under three models (no phylogeographic reconstruction, phylogeographic reconstruction without BSSVS and phylogeographic reconstruction with BSSVS). The monophyly statistic represents the posterior probability that the defined groups of sequences are monophyletic. Each defined group (Table 1) includes all C4 isolates from the countries named.

## 3 Results and discussion

### 3.1 Density and temporal dynamics of poultry

Relevant ecological data sets were collated for ROK. Estimates of livestock density were obtained for ROK (Robinson et al., 2014) and are presented in Fig. 1a and b. Chicken density is relatively homogeneous among provinces (Fig. 1a). In contrast, domestic duck densities (Fig. 1b) are noticeably higher in western mainland areas (Jeonbuk, Jeonnam, Chungnam, Chungbuk and Gyeonggi) than in eastern areas (Gyeongnam, Gangwon, Daegu, Gyeongbuk and Ulsan; see Fig. 1c for locations). Longitudinal data from commercial farms in ROK (Republic of Korea Livestock Census 2014) suggest that there are small but regular summer peaks in the number of domestic chickens in ROK. No seasonal changes in domestic duck intensity were observed in commercial farms. Although weak seasonality in domestic poultry numbers on commercial farms in ROK is unlikely to affect H5N8 dynamics, we cannot rule out seasonal variation in poultry numbers on smaller subsistence plots, as has been reported in China free-grazing duck poultry production systems (Cappelle et al., 2014).

### 3.2 Density and temporal dynamics of wild waterfowl

**Fig. 2:**
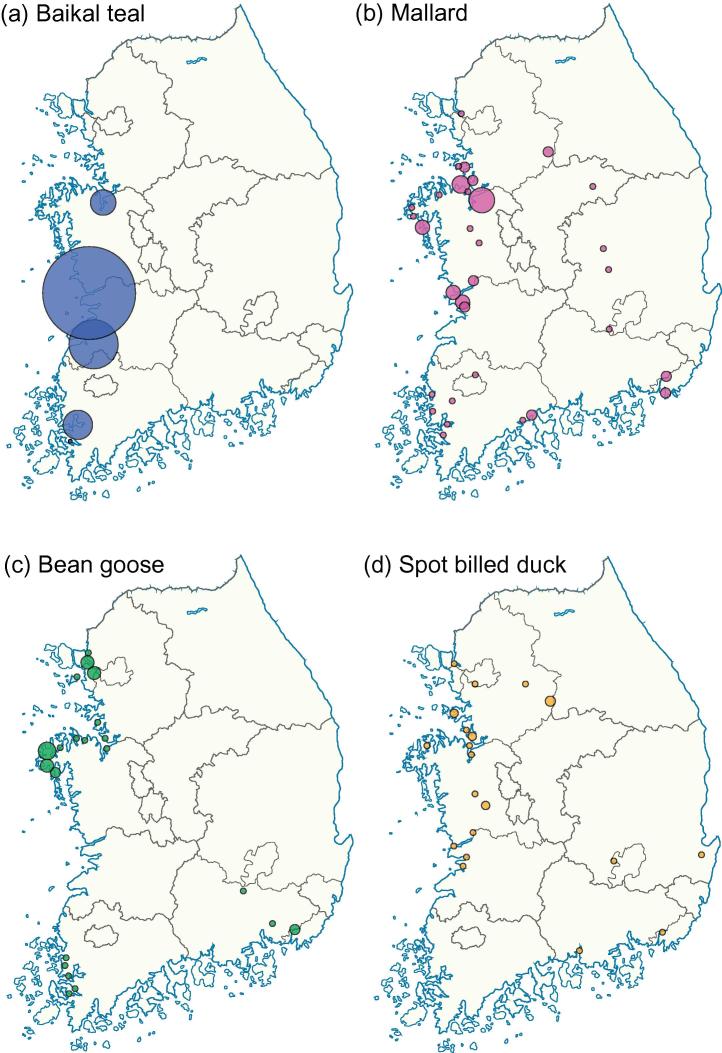
Maps generated from ROK Ministry of Environment Wild Bird Census for winter 2014 data showing the number of overwintering waterfowl for the four most common species in ROK. Circles are proportional to estimated bird numbers at sites. Geographic locations are approximate. Bird species and total observed numbers are as follows: (a) Baikal teal (*Anas formosa*) (365,641), (b) mallard (*Anas platyrhynchos*) (155,208), (c) bean goose (*Anser fabalis*) (72,225), (d) spot billed duck (*Anas poecilorhyncha*) (68,204).

**Fig. 3:**
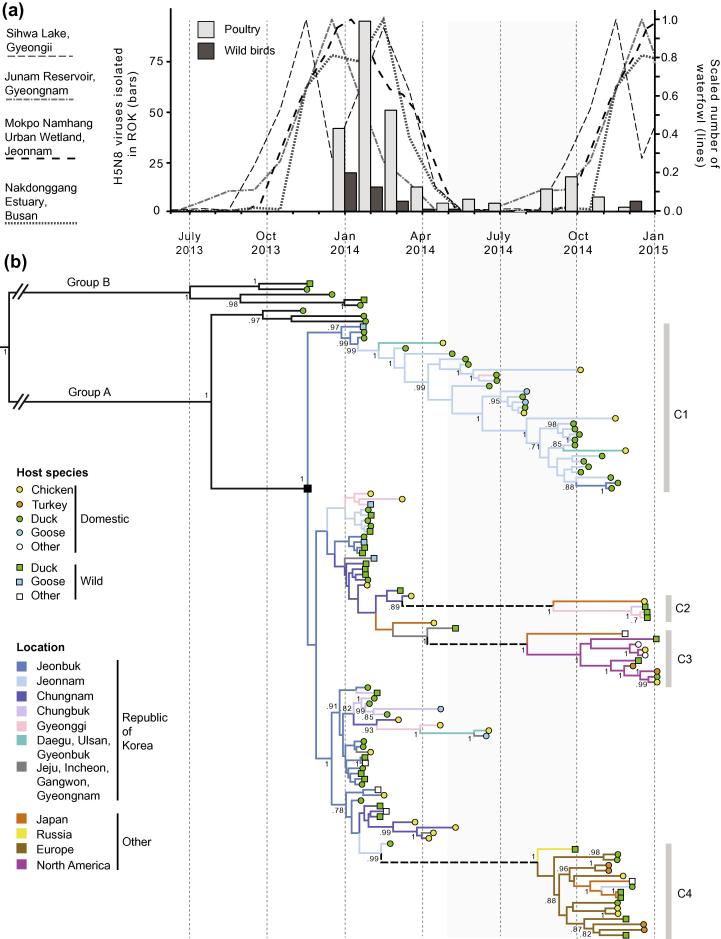
(a) Bird population dynamics in ROK. Lines represent the scaled average number of waterfowl observed at four sites across ROK (counts scaled so the maximum count at each site equals one). Bars represent the number of H5N8 viruses isolated in ROK in 2014 from wild birds (dark gray) and domestic birds (light gray). (b) The estimated MCC phylogeny of Eurasian H5N8 and American H5 strains (all H5 clade 2.3.4.4). Branch lengths represent time and the tree is placed on the same timescale as the plot in part (a) above. Branch colors represent locations inferred via discrete trait reconstruction using BSSVS (see key). “Long branches”, as discussed in the main text, are dashed. Squares and circles at tips represent host species (see key). Provinces in ROK correspond to the locations and colors used in Fig. 1. Numbers at nodes show posterior probabilities >0.7. Several sets of proximate provinces which are represented by few isolates have been grouped. A fully annotated tree is provided in Fig. A.1. (For interpretation of the references to color in this figure legend, the reader is referred to the web version of this article.)

Maps generated from the Ministry of Environment Wild Bird Census for Winter 2014 indicate that overwintering waterfowl mostly inhabit the west of ROK (Fig. 2). The most common waterfowl in winter in ROK, the Baikal teal (*Anas formosa*), occurs in high numbers only on the west coast (Fig. 2a). The other three most common waterfowl (mallard, *Anas platyrhynchos*; bean goose, *Anser fabalis*; spot billed duck, *Anas poecilorhyncha*) also mostly inhabit the west of the country (Fig. 2b–d). Counts of waterfowl show that the period of peak migration into ROK occurs between October and January each year, with the greatest number of waterfowl present in January (Fig. 3a). Due to a paucity of published raw longitudinal data our bird counts are derived from only four sites in ROK. These data therefore will not reflect the full diversity of local flyways or of migration patterns for different species. Fluctuations that reflect local conditions, such as the effects of frozen wetlands on bird density, or the effect of averaging bird migration patterns across a variety of waterfowl species, may also be present. However, annual trends in waterfowl migration into ROK are consistent among the four sites. The observation that high numbers of birds migrate to ROK to overwinter between October and March is further supported by other published longitudinal studies from which suitable raw data was not available (Hur et al., 2005; Lee et al., 2010; Nam et al., 2012; Shin et al., 2011).

### 3.3 Phylogeographic analyses of H5N8

To reconstruct the phylogenetic history and geographic dissemination of H5N8 in ROK, we performed discrete phylogeographic molecular clock analyses using BEAST. The maximum clade credibility phylogeny (Fig. 3b) strongly supports two lineages, denoted Groups A and B following the nomenclature of Jeong et al., 2014 (Jeong et al., 2014). Group B includes viruses that have a different internal gene constellation to Group A isolates. Viruses from Group A dominated the outbreaks in ROK (Jeong et al., 2014; Lee et al., 2014).

#### 3.3.1 First wave of viral entry to ROK

**Fig. 4:**
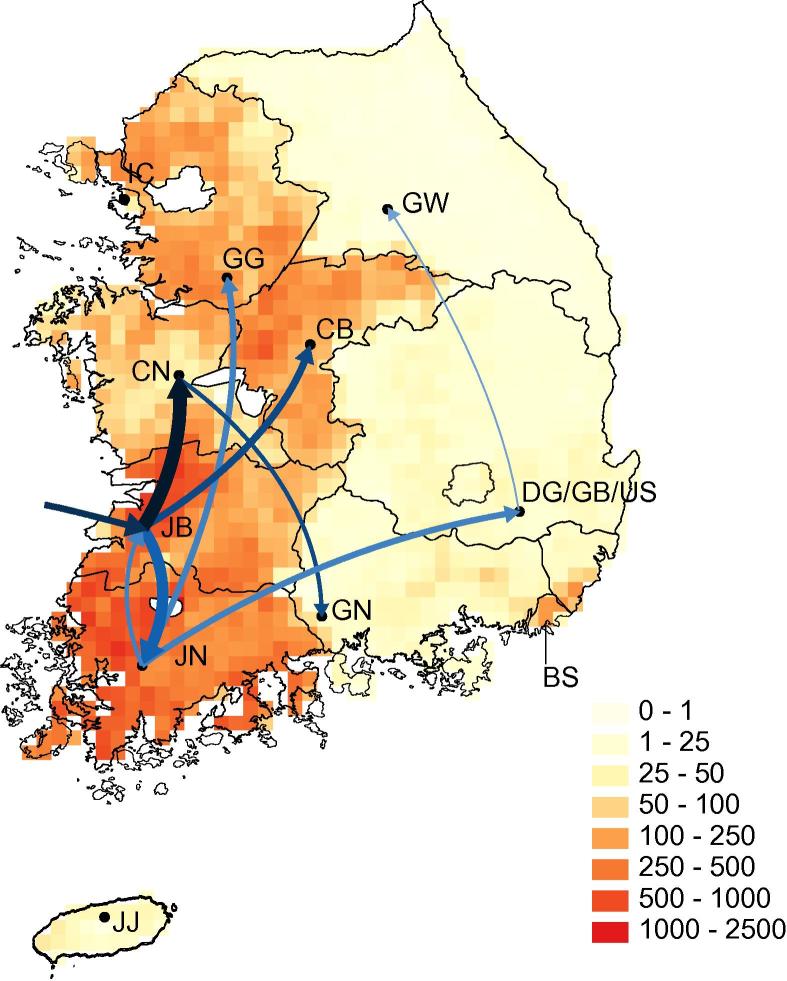
Map representing the estimated trajectory of the H5N8 spread in ROK. Arrows connecting locations represent directions of movement with Bayes Factor support >10. Arrow colors represent Bayes Factor support for rate indictors, with darker blue indicating better support. Arrow thicknesses are proportional to the inferred values of Markov jumps between locations, such that a wider arrow represents more migration between a pair of locations. Yellow and orange background show the estimated density (numbers per kilometer) of domestic ducks (colors in key). Province abbreviations are the same as those used in Fig. 1. (For interpretation of the references to color in this figure legend, the reader is referred to the web version of this article.)

Our data indicate that H5N8 first entered ROK via the province of Jeonbuk (Fig. 4). The ancestral node of all Group A Korean isolates (black square in Fig. 3b) has a high probability of being located in Jeonbuk (location posterior probability = 0.99). This result appears to be robust to potential undersampling because the basal sequences of several well-supported clades were sampled in Jeonbuk and exhibit short terminal branch lengths. Furthermore, the only two Korean sequences in Group B are also both from Jeonbuk.

Our analyses suggest that H5N8 first entered ROK at a time and in a place associated with the entry of wild waterfowl during winter migration. Specifically, the most recent common ancestor (MRCA) of the Group A sequences from ROK (black square in Fig. 3b) is estimated to have existed in Jeonbuk in mid-November 2013 (95% highest posterior density (HPD) interval = mid-October to mid-December 2013). This interval is coincident with the season during which overwintering waterfowl arrive in greatest numbers into ROK (Fig. 3a). Large numbers of wild waterfowl overwinter in Jeonbuk province, especially Baikal teal (Fig. 2). Consequently, the arrival of H5N8 to Jeonbuk is consistent with migrating wild waterfowl carrying the novel H5N8 subtype to ROK during their winter migration. That the first cases of H5N8 in both poultry and wild birds were identified near an important habitat of wild migratory birds (Donglim Reservoir; Jeong et al., 2014), supports the conclusion that wild waterfowl likely introduced H5N8 to ROK. The index cases of the November 2006 (Lee et al., 2008), April 2008 (Kim et al., 2010) and December 2010 (Kim et al., 2011) outbreaks of GsGd lineage H5N1 in ROK were also from Jeonbuk, suggesting that risk factors for the introduction of HPAI may be common to both strains.

We cannot formally exclude the possibility that this first wave of virus entry may have involved multiple introduction events to ROK, as opposed to a single introduction followed by local dissemination. However, isolates from the first wave were detected over a very short time window and are typically linked by short branch lengths in the molecular clock phylogeny (Fig. 3b), which supports a single epidemic origin. Furthermore, epidemiological reports of the spatio-temporal incidence of H5N8 in ROK support within-country transmission from a single epidemic origin in Jeonbuk to neighbouring provinces to the north and south (Jeong et al., 2014).

#### 3.3.2 H5N8 spread within ROK

Following its establishment in Jeonbuk, H5N8 spread widely across the west of ROK (Fig. 4). There is strong support for lineage migration between Jeonbuk and Jeonnam in the southwest (Bayes Factor = 141). Northwards dispersal of the virus from Jeonbuk to Chungnam and Chungbuk along the west of ROK is also well supported by the analysis (Fig. 4; Bayes Factors = 12,208 and = 269, respectively). These western provinces, among which most transmissions have occurred (Fig. 4), are characterized by high domestic duck densities and high numbers of overwintering waterfowl (Figs. 1 and 2).

By contrast, all eastern provinces (including Daegu, Ulsan, Gyeongbuk, Gangwon and Gyeongnam) are characterized by a combination of (i) few H5N8 outbreaks, (ii) little or no onward transmission, (iii) a lack of phylogenetic clustering between geographically nearby isolates, and (iv) low domestic and wild duck density. Despite being geographically proximate in the southeast of ROK, the three sequences from Daegu, Ulsan and Gyeongbuk are clearly separated from each other on the MCC tree and therefore each represents a separate virus introduction event. These results suggest that these eastern provinces may be “sink” regions, within and from which there is little or no onward transmission (Figs. 1 and 4). Five of the six isolates from these eastern provinces are from domestic chickens, which is an unusually high proportion for our dataset and reflects the lower density of *Anatidae* hosts there. We suggest that the low density of wild waterfowl and domestic ducks prevents the establishment of persistent chains of H5N8 transmission in the eastern regions of ROK.

While H5N8 appears to have persisted more in waterfowl-rich areas in the west and persisted less in waterfowl-poor areas in the east, it is difficult to determine the relative contributions of domestic ducks and wild waterfowl to this observed pattern. This is because, at the provincial level, areas of high domestic duck density and areas harboring many overwintering wild waterfowl coincide. We note that the longest persisting clade in ROK, clade C1 in Fig. 3b, was isolated almost exclusively from domestic birds in a single province over a period of more than six months. This persistence in domestic birds in a single region contrasts with non-persisting Asian outbreaks in clades C2 and C4, which were sampled from four different East Asian locations and mostly from wild birds. Thus domestic poultry appear more important in the persistence of H5N8 within ROK, whilst wild birds appear key to viral introduction to new locations.

Our observations that waterfowl densities are more important than domestic chicken density for regional emergence, persistence and dissemination of H5N8 are consistent with known pathological effects of the virus. H5N8 causes lower mortality in ducks than in chickens (Kanehira et al., 2015; Kang et al., 2015; Kim et al., 2014b; Song et al., 2014; Wu et al., 2014; Zhao et al., 2013). Infections are harder to identify in ducks than chickens because symptoms are typically less severe, so duck cases may be detected later and culled less rapidly than those in chickens. Thus waterfowl may be more effective vectors of H5N8 than chickens, congruent with our observations that waterfowl-rich areas are more important in the emergence of new outbreaks than areas rich in domestic chickens. As was noted previously in relation to GsGd lineage H5N1 prevalence (Jeong et al., 2014), the observed higher prevalence of H5N8 in ROK compared to Japan may thus be explained in part by the larger size of the domestic duck populations in ROK.

Our dataset included different numbers of sequences for each location, which has the potential to affect phylogeographic inference, particularly for undersampled locations which are more commonly inferred as sink regions. Whilst the general trend of widespread transmission in the west of ROK and rare transmission to the east of ROK appears relatively robust and is supported by epidemiological information, the exact inferred trajectory of H5N8 spread to the east of ROK appears sensitive to sampling (Fig. A.2). Larger and more comprehensive datasets would enable us to sub-sample isolates so that sample sizes are homogeneous among locations, which would allow us to definitely rule out any potential sample size effects.

#### 3.3.3 Proposed second wave of virus transmission

Internal branches in the molecular clock phylogeny (Fig. 3b) that represent H5N8 transmission within ROK following its introduction in 2014, are generally short and almost all are of less than 8 weeks duration (Fig. 3b). However, three long branches are notable outliers from this pattern, each representing an internal branch of more than 17 weeks duration (dashed branches in Fig. 3b). The sequences from ROK (and Japan) that are descended from these three long branches were all sampled between 18th November and 19th December 2014.

There are two interpretations of these long branches, depending on whether the phylogeographic results are interpreted naively, or if they are interpreted in the context of known bird ecological data. Considered naively, the long branches would represent unsampled persistence of H5N8 within ROK during summer 2014. If so, then coincident sampling of these lineages in late 2014 requires a specific explanation (for example, a sudden increase in sampling at that time, which identified multiple infections that had been overlooked during the summer). However, evidence for the persistence of clade C1 in domestic ducks in Jeonnam throughout the summer of 2014 indicates that continuous sampling was undertaken, at least in parts of ROK. We suggest that this ecologically uninformed interpretation is implausible, and we favour an alternative that is informed by the ecological data. It is important to note that each long branch spans a period when few waterfowl are present in ROK (gray shading in Fig. 3b), yet the tips of these long branches were sampled during the period of peak inward migration of overwintering waterfowl. It is therefore much more likely that these clade C2 and C4 strains represent re-introductions of H5N8 into ROK via wild birds, and do not result from cryptic or unsampled persistence of H5N8 within ROK. Hence the long branches most likely represent transmission from an unsampled reservoir. Existing satellite tracking, GPS and modeling data suggest that migratory waterfowl typically migrate to or via ROK from sites in northern or northeastern China, Russia and Mongolia (Takekawa et al., 2010; Tian et al., 2015; Verhagen et al., 2015b), so the unsampled reservoir may be located in one of these regions. This interpretation implies at least three separate introductions of H5N8 to Asia in late 2014 from an as-yet uncharacterized source as part of a ‘second wave’ of virus entry.

The same model of transmission from an unsampled reservoir can explain the long-branches leading to North American samples (denoted clade C3 on Fig. 3b) and European and Russian samples (denoted clade C4 on Fig. 3b) from the same time period. The long internal branch that leads to clade C4 (Fig. 3b) contains viruses isolated in Russia, ROK, Japan and Europe. The presence of isolates from West Europe, Russian and East Asia in this clade is very striking, especially given the large geographic distances between these locations. Careful study of clade C4 shows that the phylogenetic topology within it is not certain, as evidenced by overlapping TMRCAs for several internal nodes in C4 (Table 1 and Figs. A.3–A.5) and low posterior support for many nodes within the clade (Fig. 3b). There is a very low probability that all the European isolates within C4 form a strictly monophyletic cluster (Table 1), whereas there is greater statistical support for the grouping of three Asian samples with three European samples, to the exclusion of other European isolates (Fig. 3b and Table 1).

These results leave open two possible scenarios. In the first, there is a single introduction of C4 viruses into Europe (from an unsampled reservoir location) followed by rapid viral lineage movement from Europe to East Asia. In the second scenario, C4 viruses were independently dispersed from an unsampled reservoir to Western Europe and East Asia during July to September 2014. Because the European samples do not form a strictly monophyletic clade, this second scenario requires there to have been at least two, and possibly many more, separate introductions of C4 viruses into Europe from the reservoir population. Based on the patterns observed in ROK, we again posit that this may represent long distance transmission via migratory wild birds from an unsampled reservoir. This hypothesis follows recent suggestions that the almost simultaneous detection of H5N8 across the world could be a result of birds carrying the virus from unsampled breeding grounds in Russia and Beringia (Lee et al., 2015; Verhagen et al., 2015a,b). Ring recovery data from wild ducks suggests that intercontinental transmission by wild birds is plausible (Verhagen et al., 2015b). If this model is correct, then the phylogenetic data can only be reconciled if H5N8 entered Europe on at least two separate occasions, and entered Asia at least three times.

## 4 Conclusions

In this study we investigated the factors underlying the emergence and persistence of H5N8 HPAI in the Republic of Korea by analyzing new and previously available viral gene sequences using molecular clock and phylogeographic methods, and by interpreting the results in the context of data on avian ecology. We suggest two separate waves of migrating wild waterfowl contributed to the presence of H5N8 HPAI in ROK; the first during its initial emergence in Asia in 2013/2014, and the second during late 2014. We find that H5N8 initially emerged in ROK in an area of high wild bird and domestic duck density, at a time associated with the migration of overwintering wild waterfowl into the region. Although we cannot formally exclude the effect of climatic variables such as temperature, which are naturally co-linear with bird migration patterns, these data suggest that migrating wild waterfowl were important in the establishment of H5N8. Despite several introductions, the virus did not become established in the east of ROK, which is an area characterized by low numbers of domestic ducks and wild waterfowl. Domestic duck distribution appears to have been important in regional persistence. We posit a model of a second wave of virus introductions by wild birds from an unsampled reservoir population, which may also explain the observed long distance transmissions to Europe, North America and Russia. The second wave of H5N8 introduction is supported by the presence of long-branches in the tree that coincide with the absence of wild bird migration to ROK.

Our results support recent hypotheses that wild waterfowl and domestic ducks are important in the emergence and maintenance of HPAI H5N8 (Verhagen et al., 2015a,b). We highlight the importance of interpreting phylogeographic analyses in the context of available ecological data, especially when sample sizes are small or when key locations are not sampled. Future studies formally integrating phylogenetic data with ecological data should be conducted in order to more clearly identify factors involved in H5 HPAI emergence and to help target surveillance resources to high-risk source areas. Publication of the exact geographic locations of viral isolates and collation of existing ornithological datasets would allow outbreaks to be considered in their proper ecological context. Determining the ecological drivers of HPAI transmission will become increasingly important for both human and animal health as domestic poultry production continues to intensify across the world (Jones et al., 2013).

## Figures and Tables

**Table 1:** Phylogenetic statistics for various groups of isolates within clade C4.

Groups of isolates	Phylogeographic model, with BSSVS		Phylogeographic model, without BSSVS		No phylogeographic model	
	Monophyly probability	TMRCA⁎ (95% HPD interval)	Monophyly probability	TMRCA⁎ (95% HPD interval)	Monophyly probability	TMRCA⁎ (95% HPD interval)
Japan and Korea	1.00	0.21 (0.15, 0.27)	1.00	0.21 (0.15, 0.27)	1.00	0.22 (0.16, 0.29)
Europe	<0.001	0.34 (0.26, 0.44)	<0.001	0.35 (0.26, 0.44)	<0.001	0.37 (0.28, 0.457)
Russia and Europe	<0.01	0.38 (0.29, 0.47)	<0.01	0.38 (0.30, 0.46)	<0.01	0.38 (0.30, 0.464)
Europe, Japan and Korea	0.62	0.34 (0.26, 0.44)	0.55	0.35 (0.26, 0.44)	0.28	0.37 (0.28, 0.46)
Russia, Japan and Korea	0	0.38 (0.29, 0.47)	0	0.37 (0.23, 0.46)	0	0.37 (0.29, 0.46)

BSSVS = Bayesian stochastic search variable selection.

⁎ Years before 30th December 2014.
