# Supplementary figures and images for: Wild waterfowl migration and domestic duck density shape the epidemiology of highly pathogenic H5N8 influenza in the Republic of Korea
Source: Infect Genet Evol. 2015 Aug;34:267–77. doi: 10.1016/j.meegid.2015.06.014 (PMC4539883; doi:10.1016/j.meegid.2015.06.014)

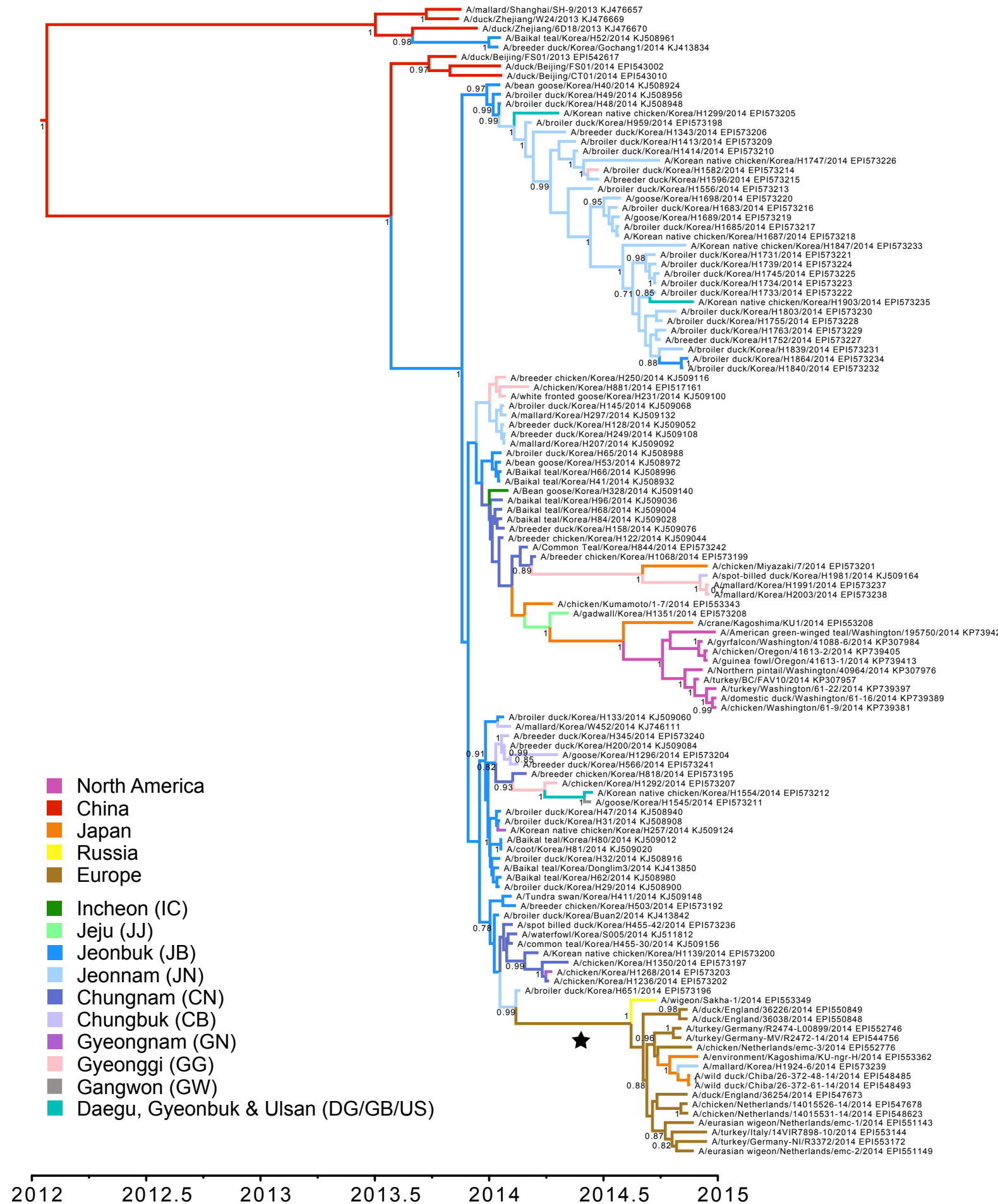

Supplement: Supplementary Fig. A.1 [file mmc1.pdf]
