# Supplementary material for: Wild waterfowl migration and domestic duck density shape the epidemiology of highly pathogenic H5N8 influenza in the Republic of Korea
Source: Infect Genet Evol. 2015 Aug;34:267–77. doi: 10.1016/j.meegid.2015.06.014 (PMC4539883; doi:10.1016/j.meegid.2015.06.014)
Supplement: Supplementary Fig. A.2 [file mmc2.pdf]

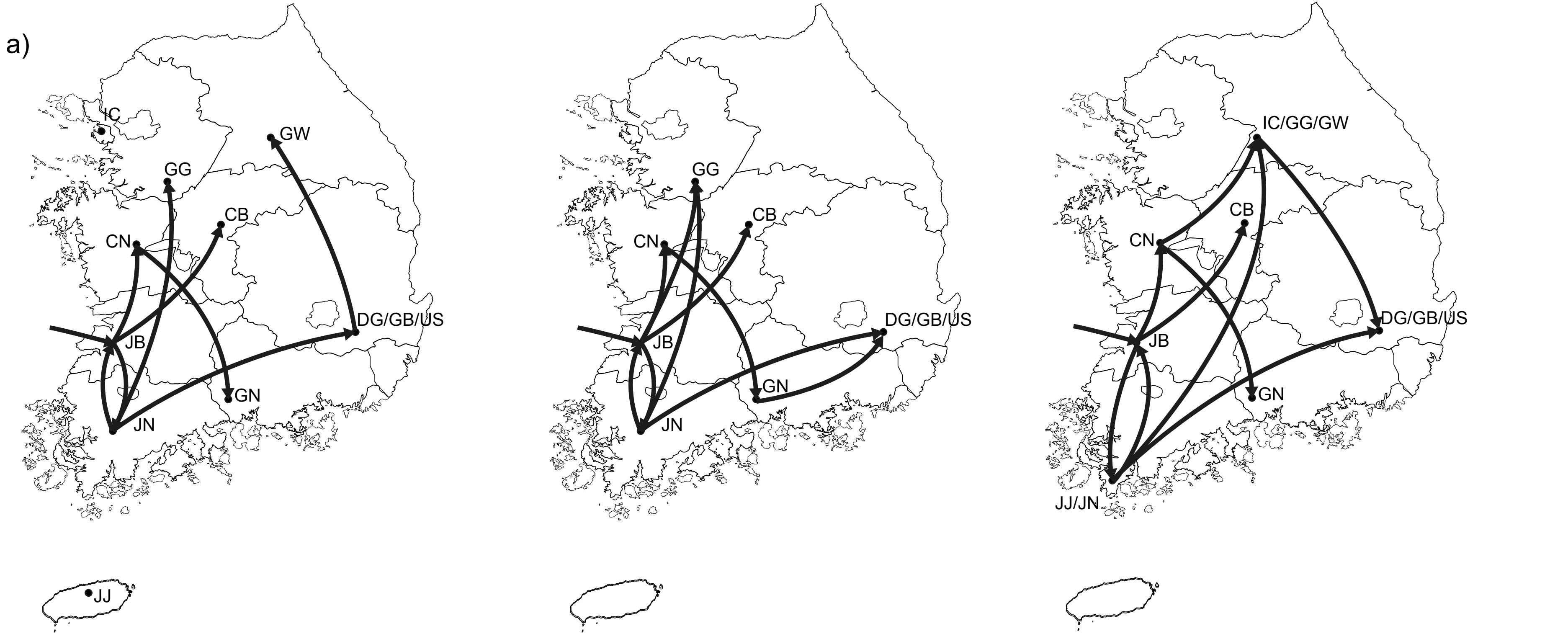

Fig. A.2. Effect of different sampling and grouping of provinces with only one sequence on phylogeographic inference. Arrows show inferred epidemiological links where Bayes Factor support for rate indictors >10, as determined using SPREAD. a) Results as shown in Figure 3. b). Sequences isolated in provinces from which only one sequence is available have been removed from the alignment (except DG/GB/US, which have been grouped). c) Sequences isolated in provinces from which only one sequence is available have been grouped with geographically neighbouring provinces for phylogeographic inference.
