# Supplementary material for: Wild waterfowl migration and domestic duck density shape the epidemiology of highly pathogenic H5N8 influenza in the Republic of Korea
Source: Infect Genet Evol. 2015 Aug;34:267–77. doi: 10.1016/j.meegid.2015.06.014 (PMC4539883; doi:10.1016/j.meegid.2015.06.014)
Supplement: Supplementary Fig. A.3 [file mmc3.pdf]

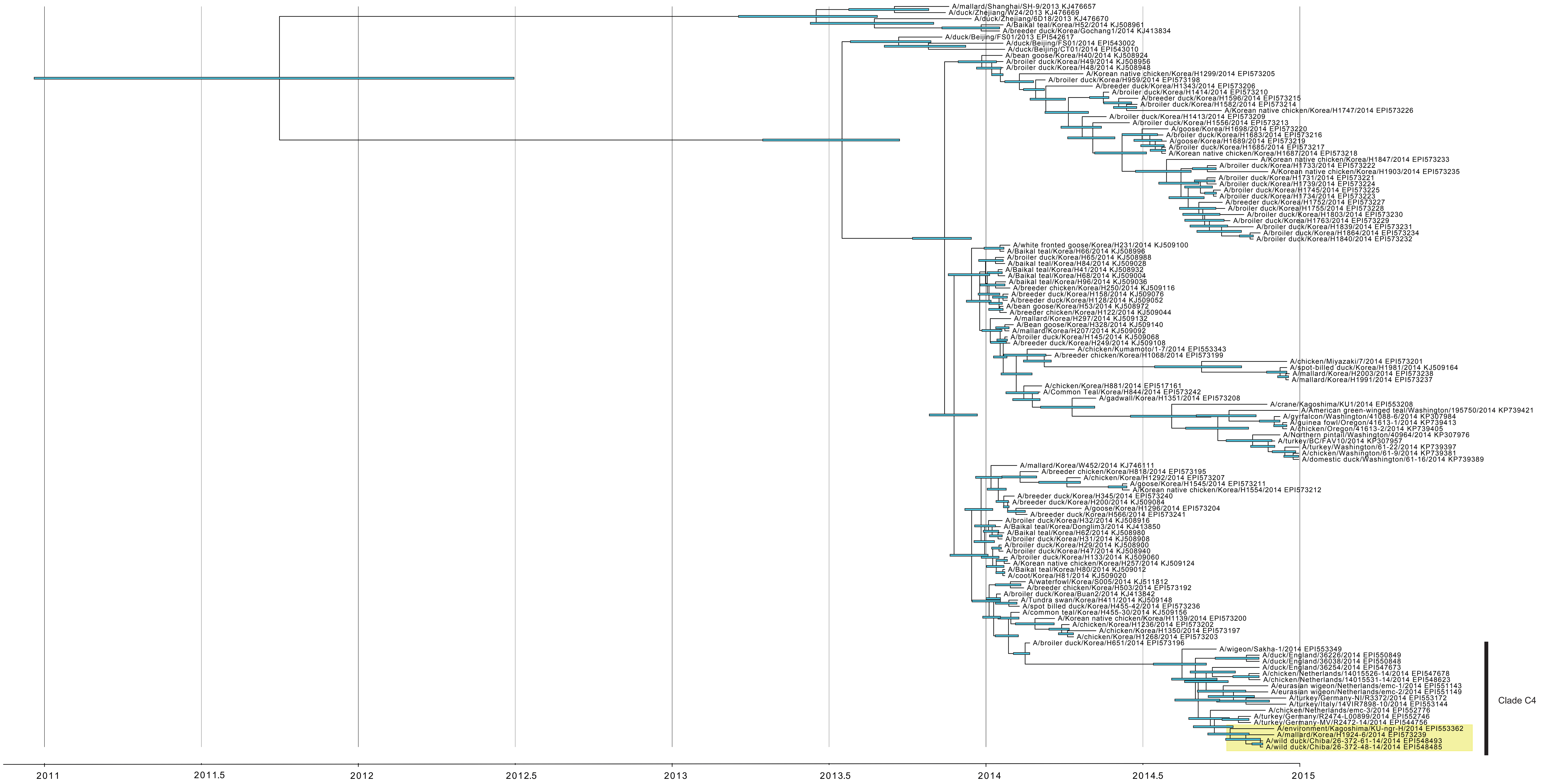

Figure A.3. Maximum clade credibility tree for reconstruction without phylogeographic model. Yellow box shows ‘Japan and Korea’ clade in which monophyly is strongly supported (Table 1). Note that, although A/wigeon/Sakha-1/2014 appears to be basal to clade C4 is this MCC tree, this position is not well supported by monophyly statistics (monophyly statistic 0.28), which indicates the Russia sequence often occurs internally the the C4 clade.
