# Supplementary material for: Wild waterfowl migration and domestic duck density shape the epidemiology of highly pathogenic H5N8 influenza in the Republic of Korea
Source: Infect Genet Evol. 2015 Aug;34:267–77. doi: 10.1016/j.meegid.2015.06.014 (PMC4539883; doi:10.1016/j.meegid.2015.06.014)
Supplement: Supplementary Fig. A.4 [file mmc4.pdf]

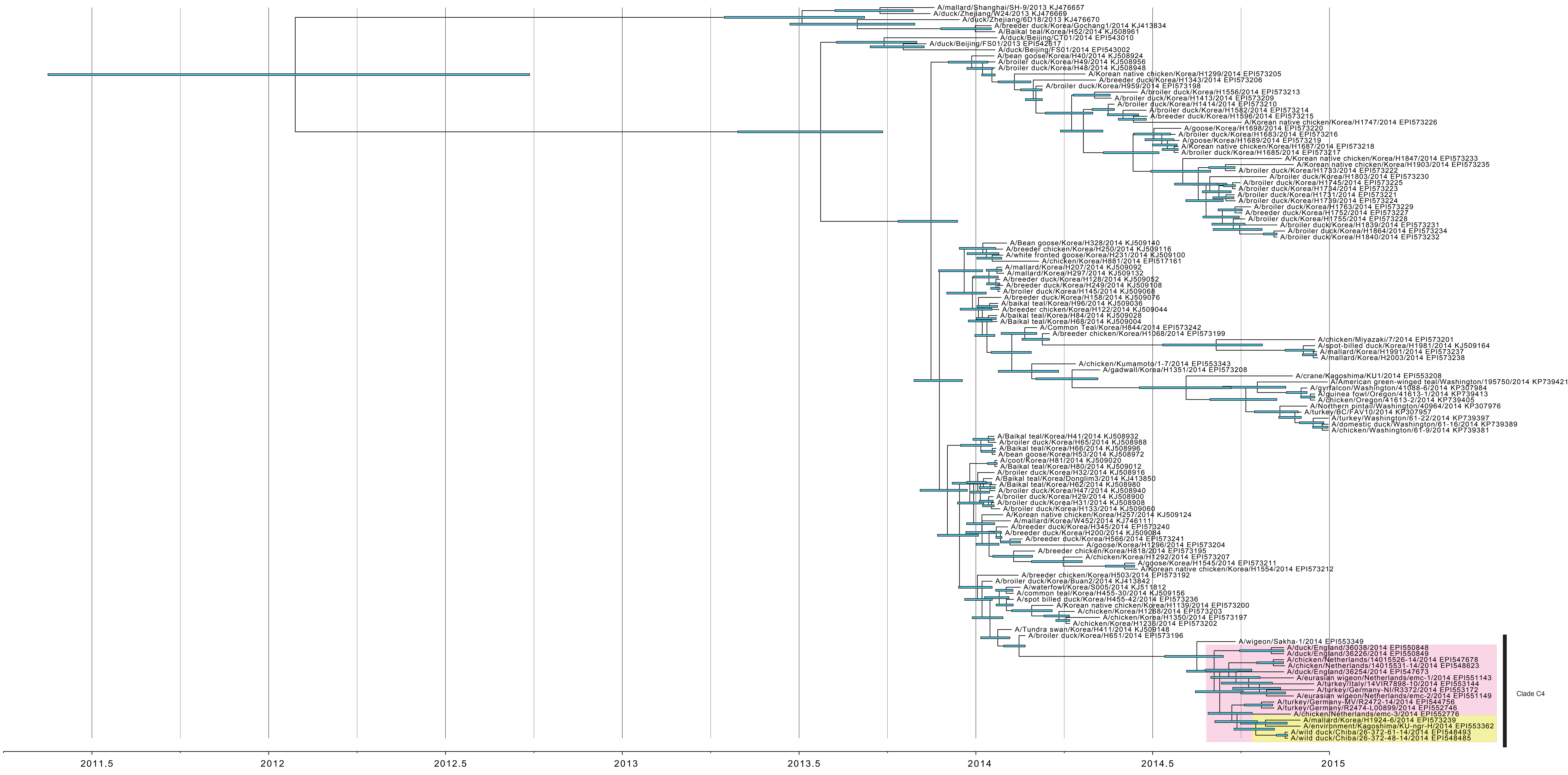

Figure A.4. Maximum clade credibility tree for reconstruction with phylogeographic model and without BSSVS. Yellow box shows 'Japan and Korea' clade in which monophyly is strongly supported (Table 1). Pink box shows 'Japan, Korea and Europe' clade (Table 1). Monophyly of this clade is only very weakly favoured by this model (monophyly statistic 0.55).
