# Supplementary material for: Wild waterfowl migration and domestic duck density shape the epidemiology of highly pathogenic H5N8 influenza in the Republic of Korea
Source: Infect Genet Evol. 2015 Aug;34:267–77. doi: 10.1016/j.meegid.2015.06.014 (PMC4539883; doi:10.1016/j.meegid.2015.06.014)
Supplement: Supplementary Fig. A.5 [file mmc5.pdf]

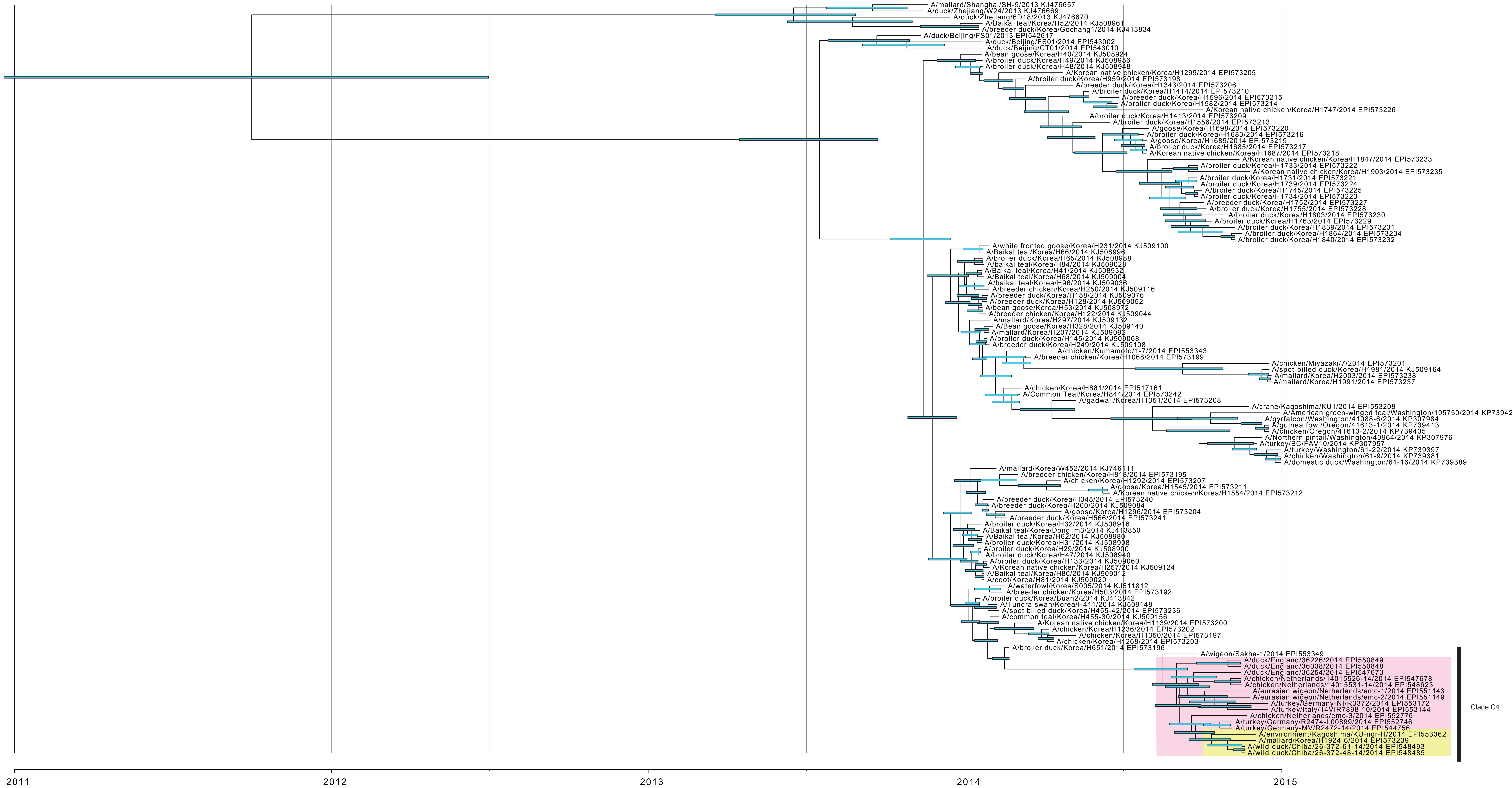

Figure A.5. Maximum clade credibility tree for reconstruction with phylogeographic model and with BSSVS. Yellow box shows ‘Japan and Korea’ clade in which monophyly is strongly supported (Table 1). Pink box shows ‘Japan, Korea and Europe’ clade (Table 1). The monophyly statistic for this clade (0.62) only very weakly supports consistent monophyly during tree estimation.
